# Characteristics and liming potential of biochar types from potato waste and pine-bark

**DOI:** 10.1371/journal.pone.0282011

**Published:** 2023-02-21

**Authors:** Samukelisiwe P. Vilakazi, Pardon Muchaonyerwa, Nkosinomusa N. Buthelezi-Dube

**Affiliations:** School of Agriculture, Earth and Environmental Sciences, University of KwaZulu-Natal, Scottsville, Pietermaritzburg, South Africa; Universiti Teknologi Petronas: Universiti Teknologi PETRONAS, MALAYSIA

## Abstract

Large amount of wastes are burnt or left to decompose on site or at landfills where they cause air pollution and nutrient leaching to groundwater. Waste management strategies that return these food wastes to agricultural soils recover the carbon and nutrients that would otherwise have been lost, enrich soils and improve crop productivity. This study characterised biochar produced by pyrolysis of potato peels (PP), cull potato (CP) and pine bark (PB) at 350 and 650°C. The biochar types were analysed for pH, phosphorus (P) and other elemental composition. Proximate analysis was done following ASTM standard 1762–84, while surface functional groups and external morphology characteristics were determined using FTIR and SEM; respectively. Pine bark biochar had higher yield and fixed carbon (FC), and lower ash content and volatile matter than biochar types from potato wastes. The liming potential of CP 650°C is greater than that of PB biochars. Biochar types from potato waste had more functional groups even at high pyrolysis temperature relative to pine bark. Potato waste biochars showed an increase in pH, calcium carbonate equivalent (CCE), K and P content with increasing pyrolysis temperature. These findings imply that biochar from potato waste may be valuable for soil C storage, remediating acidity and increasing availability of nutrients especially K and P in acidic soils.

## Introduction

Over 30% of food produced across the world goes to wastes [1], which constitutes approximately 1.3 billion tonnes [2]. These food wastes end up in landfills where they undergo a series of bioconversions into biogas [3]. When disposed of at landfills, they can result in pollution of ground water from leaching of nitrates while the gases produced at such sites particularly ammonia, methane, carbon dioxide (CO_2_) and nitrous oxides (N_2_O) and odours contribute to poor air quality and increase concentration of greenhouse gases in the atmosphere [4]. Potato wastes are among the major food wastes globally.

A total of 35.5, 3.84 and 1.09 million tons of potato waste are produced globally, in Africa and in South Africa, respectively [5]. These waste materials have little economic value, with only a portion used as animal feed [6]. A large proportion of the waste is disposed at landfills sites where they produce odours during anaerobic decomposition while the nutrients mineralised may leach to groundwater [7]. While potato peels can be used for production of biogas and extraction of lactic acids, phenolic acids and alkaloids which can contribute to food and pharmaceutical industries [6], the high costs limit the practical benefits of such industrial uses of potato wastes. Waste management strategies that return these food wastes to agricultural soils could be a cheaper option that enriches soils and improve crop productivity.

Potato tubers contain substantial concentrations of nitrogen (N) [8] and potassium (K) [9], and the addition of potato wastes to soils could improve crop productivity in the long-term [7]. Potato peel wastes were reported to contain 40% C, 1.4% N, 3.09% K, 0.3% P, 0.156% calcium (Ca), 0.150% magnesium (Mg) and 0.041% sodium (Na) [10]. Olsen et al [7] also reported that cull potato (potatoes for disposal) contained 2.14, 2.40, 0.29, 0.074, 0.148 and 0.0029% of N, K, P, Ca, Mg and Na, respectively. Both wastes have high concentrations of macronutrients, particularly N and K. The two studies suggested differences in elemental composition between cull potatoes and potato peels, although the differences could partly be because the wastes were produced under different conditions in different parts of the world. The elemental composition of potato culls and peels and its effect on biochar characteristics has not been thoroughly studied. This knowledge is vital when deciding between the direct uses of potato feedstocks or further processing into biochar as strategies for recycling of nutrients and storing carbon in the soil.

Conversion of waste biomass to biochar has been a promising approach to reducing waste disposal challenges [11] and improving nutrient recycling [12]. The pyrolysis of plant-based materials to biochar has been prominent in the literature, particularly rice husk, straw, maize, and pine bark [13–15]. The biochar types from these materials have been found to contain high aromatic C assignable to their lignin content [16], which prompts high resistance to microbial decomposition. The high C content with aromatic structure gives biochar the ability to sequester C in the soil, which could otherwise have been emitted to the atmosphere as CO_2_-C. In South Africa, pine bark from the forestry industry, is one of the major plant-based waste materials. While characteristics of biochar from pine bark have been shown in numerous reports [17, 18], there is a paucity of information on the characteristics of biochar derived from potato waste. The differences in the chemical composition of these two plant materials may influence their biochar characteristics, and there is need to understand how characteristics of potato wastes biochar compare with that from woody material (pine bark), one of the most used materials. The most important parameters used for biochar characterisation include contents of volatile matter (VM), ash, and fixed carbon (FC).

Biomass materials with high nutrient concentration can produce biochar with high ash content and liming ability [19], which make such biochar potentially useful in remediation of acidic soils. The ash and FC can predict biochar behaviour in terms of nutrient supply, liming potential, and nutrient retention [20]. In addition to feedstock types, the characteristics of biochar also depends on pyrolysis temperatures. Biochar produced at low temperature has been found to have higher VM and yield and lower FC than those produced at high temperature [21]. Higher pyrolysis temperature also increases surface area and porosity, which increases surface adsorption capacity while the higher aromatic C content increases the recalcitrance of the biochar [21–24]. While numerous studies have been conducted on characteristics of biochar types from other waste products, there is a gap in knowledge on characteristics of biochar from potato wastes, such as yield, VM, FC, aromatic C content, surface functional groups, physical structure, and nutrient composition. Understanding the characteristics of potato waste biochar is essential in establishing the potential contribution of the material in sequestering C (stabilisation), providing and retaining nutrients when added to agricultural soils. Therefore, the objective of this study was to determine the effect of feedstock and pyrolysis temperature on the physico-chemical characteristics and liming potential of biochar produced from potato peels (PP), cull potatoes (CP) and pine bark (PB).

## Materials and methods

The study was conducted at the University of KwaZulu-Natal (UKZN) Pietermaritzburg campus (29° 37’ 33.9” S; 30° 24’ 14’E) in the province of KwaZulu-Natal in South Africa.

### Potato and pine bark wastes and their characteristics

The biochar used in this study was produced from potato peels (PP), cull potatoes (CP) and pine bark (PB). The PP were freshly collected in the Pietermaritzburg CBD from shops that use potatoes for production of fresh chips. The PP were collected into black plastic bags, air-dried for seven days in a glasshouse to speed up drying and then stored in plastic bags. The CP, which consisted of whole potatoes of low market value generally rotten (were kept for up to three days), were collected from the Pietermaritzburg Fresh Produce Market located in Mkondeni, Pietermaritzburg. The CP were collected into black dustbins, chopped using a slasher and air-dried for seven days, then stored in plastic bags. The PB was collected from a private forestry by-product factory located at Cramond, Pietermaritzburg, and were kept for over a month before sampling and were air dried for seven days and stored in plastic bags for up to seven days before they were ground. The PP, CP and PB samples were ground to < 2 mm using a grinding mill machine, Retsch KG 5657 HAAN, West Germany model, and stored in white plastic bags. The particles were then oven-dried at 80°C for 24 hours before characterisation. The PB used had higher total C and FC and lower VM, total N, H, H/C and ash than potato waste (Table 1). Potato waste had higher pH, P, Mg and CEC than pine bark. The PP had higher FC, total N, ash, Ca and Mg, pH and lower VM, total O and O/C and total P than CP, while there were no differences between the potato wastes in terms of total C, total H, H/C and CEC (Table 1).

**Table 1:** Characteristics of raw feedstocks.

Feedstocks	Units	Cull potatoes	Peel potatoes	Pine bark
**Ultimate analysis**				
Total C	%	39^a^	38.9^a^	50.9^b^
Total N	%	1.10^b^	1.47^c^	0.249^a^
Total H	%	6.31^b^	6.09^b^	5.15^a^
O	%	49.1^c^	45.8^b^	43.3^a^
H/C		0.161^b^	0.156^b^	0.101^a^
O/C		1.26^c^	1.18^b^	0.85^a^
**Proximate analysis**				
Moisture content	%	11.6^b^	10.1^a^	10.3^a^
Volatile matter	%	78.3^c^	74.4^b^	70.1^a^
Fixed Carbon	%	17.3^a^	18^b^	29.5^c^
Ash	%	4.40^b^	7.63^c^	0.436^a^
**Other Characteristics**				
pH		8.12^b^	8.61^c^	4.0^a^
P	mg kg	81.9^c^	43.5^b^	14.1^a^
Ca	Cmolc kg^-1^	1.11^a^	1.66^b^	1.24^ab^
Mg	Cmolc kg^-1^	6.38^b^	9.67^c^	2.29^a^
CEC	Cmolc kg^-1^	57.7^b^	57.5^b^	12.3^a^

*Values on the same row with similar letters indicate a non-significant difference (p<0.05) and with different letter indicates a significant difference (p<0.05). The letters “ab” indicate that the mean is not significantly different from those with the letter “a” and those with the letter “b”. CP = cull potato waste; PP = peel potato waste; PB = pine bark waste. CEC = cation exchange capacity.

### Biochar production

The milled samples were pyrolysed in a muffle furnace where the temperature used was 350°C and 650°C at a rate of 10°C /min [17]. The feedstocks were pyrolysed for 2 hours per set temperature and the biochar types were cooled and weighed to determine the yield and stored in sealed plastic containers for further analysis [18].

### Volatile matter, ash content, moisture content and fixed carbon

Proximate analysis of the materials was done following the standard procedure [25]. Moisture content was determined by oven-drying the milled samples at 105°C for 2 hours, while the volatile matter was based on weight loss at 950°C for 6 min in an inert muffle furnace (Labcon, type RM 4). The ash content was determined after combustion at 750°C for 6 hours in an inert muffle furnace and fixed C was calculated by subtraction of ash (%) and volatile matter (%) from 100% [14].

### Selected physico-chemical properties of the biochar types

The pH was determined in water and KCl at a ratio of 1:10 [17]. Total C and N were analysed by dry combustion using the Leco Trumac (CNS) autoanalyser instrument [26]. Total H was analysed using the CHN elemental analyser. Total O was calculated using Eq 1 [17]. Extractable P was determined calorimetrically following AMBIC– 2 extractions [27] and analysed by the molybdenum-blue method [28]. The cation exchange capacity (CEC) was determined as the concentration of NH_4_^+^ retained after leaching with several portions of ethanol by using the Thermo Scientific Gallery Discrete Auto-analyser, and exchangeable bases were extracted using the 1M ammonium acetate (NH_4_OAC) method [29] and analysed using Atomic absorption spectrometry (Ca^2+^ and Mg^2+^) and Atomic flame spectrometry (K^+^). The biochar liming potential (calcium carbonate equivalent) was evaluated following the method by Singh et al.[30]. The recorded results were used to calculate the calcium carbonate equivalent following Eq 2.


TotalO(%)=100–(C+H+N+ASH) Eq 1

CaCO3equivalent(%)=M×(b−a)×10^(−3)×100.09×1002×W Eq 2

Where:

“M” is the molarity of NaOH (mol L^-1^), “b” is the NaOH volume (ml) used by the blank, and “a” is the volume (ml) of NaOH used by the biochar sample. The “W” is the mass (g) of biochar used.

### Surface functional groups

The Fourier Transformed Infrared (FTIR) spectra were scanned using Perkin Elmer FTIR spectrometer (FTIR-100) in the region of 400–4000 cm^-1^ of the biochars as explained by Koetlisi and Muchaonyerwa [18]. The samples were finely ground and allowed to pass through the ATR for analyses.

### External morphology and surface characteristics

The surface characteristics of the biochars were analysed by using Scanning Electron Microscope (SEM) (EVO LS15, Carl Zeiss Microscopy, New York, USA). The samples were held onto an adhesive carbon tape on an aluminium stub, sputtered with gold coating for 6 runs before viewing using a gold sputtering machine (Quorum Q150R ES, Quorum Technologies, East Sussex, UK).

### Incubation in soil for determination of liming potential

#### Soils

The two soils used were collected from the UKZN Research Farm, Ukulinga (29° 39′ 33.9″ S; 30° 24′ 14″E), and Bulwer (29° 48′ 27″ S; 29° 45′ 35″E). The Ukulinga area receives a mean annual precipitation of 750 mm and the soil was under natural vegetation. The Bulwer area receives a mean annual precipitation of 877 mm and the soil was used for cultivation of maize. The soil from Ukulinga was Bonheim form and Bulwer was a Clovelly soil form [31], which translated to Luvisol and Ferralsol, respectively, according to the IUSS-working group WRB [32]. Bulk soil samples were collected from the 0–20 cm depth, mixed and homogenized, air-dried and sieved (< 2mm) before analysis. The clay content was estimated using Mid-Infrared reflectance ((HTS-XT, Bruker, Germany). Summary characteristics of the soils are shown on Table 2. The Luvisol had 39% clay, pH_KCl_ 4.67, and C/N 16, while Ferralsol had 23% clay, pH_KCl_ 3.97_,_ and C/N 13. The Luvisol had generally higher Ca and Mg and lower total C and N, extractable P, and exchangeable K and acidity than the Ferralsol.

**Table 2:** Selected physico-chemical properties of the soil used.

Property	Luvisol	Ferralsol
pH_(KCl)_	4.67	3.97
pH_(H2O)_	5.87	4.71
Carbon (%)	4.45	5.4
Nitrogen (%)	0.268	0.403
C/N	16	13
Clay (%)	39	23
Bulk density (g cm^-3^)	1.29	1.12
Extractable P (mg/kg)	2.73	18.6
Exchangeable K (cmol_c_/kg)	0.0627	0.338
Exchangeable Ca (cmol_c_/kg)	2.24	1.02
Exchangeable Mg (cmol_c_/kg)	2.24	0.577
Exchangeable acidity (cmol_c_/kg)	1.6	8

#### Experimental set-up

The experiment was a 2×4 factorial set up in a completely randomized design, with two soils and four biochars from CP and PB pyrolysed at 350 and 650°C, in triplicates. The biochar was added as lime at the recommended rate (based on the CCE) with unamended soil as the control. The recommended lime required to neutralise acidity was calculated (Eq 3) following Manson et al.[33], and were 5 and 29 t ha^-1^ for Luvisol and Ferralsol soil, respectively. Lime (CaCO_3_) was also included as a positive control. The moisture content of the soils at field capacity was measured using a pressure plate at -33 kPa [34]. The soil (100g) and biochar were placed in 500 ml plastic containers and mixed thoroughly before moisture adjustment to 100% field capacity. The soil moisture content was corrected based on weight loss. The soils were incubated for 10 days in a constant temperature room (25°C) and analysed for pH [30].


LimeRequirement(tha−1)=["exch.acidity"‐("totalcations"xPAS/100)]xF Eq 3

Where PAS is the permissible acid saturation for the crop selected and was taken as 5% acid tolerance, for this study. The F is a factor indicating the amount of lime required to neutralize 1 cmolc L^-1^ of exchangeable acidity.

### Analysis

Total C and N, extractable P and exchangeable bases (Ca, Mg, and K) in the soils were analysed as detailed for biochar characteristics. Soil pH was determined at 1:5 soil: solution ratio, in water and 1M KCl using a pH meter (Ohaus starter 2100). Exchangeable acidity was extracted with 1 M KCl, and titrated with 0.1 M NaOH with phenolphthalein indicator [35].

### Statistical analysis

The data on characteristics and calcium carbonate equivalents of biochar types were subjected to two-way analysis of variance (ANOVA), to show pyrolysis temperature and feedstock type effects, using GenStat 18^th^ edition. A two-way ANOVA was carried out to assess the acid neutralisation potential of the biochar types in the two soils. Mean separation was done using least significant difference (LSD) at p< 0.05. The Tukey-Kramer test was also used to separate treatment means at p < 0.05 and was used when describing the results.

## Results

### Biochar yield, proximate and ultimate analysis

Biochar yield decreased with increasing pyrolysis temperature for all three feedstocks (Table 3). Biochar yield was greater for PB followed by PP with CP having the lowest at both pyrolysis temperatures (Table 3). Potato waste biochars had higher moisture content than those from PB at both pyrolysis temperatures, although there were no consistent differences between pyrolysis temperatures, with PB biochar produced at 650°C having the lowest of all the treatments (Table 3). Higher pyrolysis temperature decreased (p<0.05) volatile matter and increased ash and fixed C for all the feedstocks (Table 3). The trends were in the order: PP > CP >PB for ash and PB>CP>PP for fixed C at both pyrolysis temperatures. At 350°C the VM was significantly higher in PB biochar than the other two, while at 650°C, the biochar from PB had the lowest. Ash concentration was considerably higher while fixed C was lower for the potato biochars compared to that from PB. Differences in fixed C were rather small between biochars at 350°C, while at 650°C fixed C was higher for pine bark.

**Table 3:** Yield and proximate analysis results of the biochars.

Pyrolysis	Feedstocks	Yield	Moisture	Volatile	Ash	Fixed
Temperature			Content	Matter	Content	Carbon
(°C)		(%)	(%)	(%)	(%)	(%)
350	CP	30.1^c^	3.51^bc^	33.39^e^	10.73^c^	55.88^b^
	PP	33.9^d^	4.15^c^	29.87^d^	19.18^e^	50.95^a^
	PB	52.2^e^	2.58^ab^	41.05^f^	0.92^a^	58.03^c^
650	CP	21.8^a^	6.08^d^	14.45^c^	15.48^d^	70.08^e^
	PP	24.5^b^	4.40^c^	13.04b	26.35^f^	60.64^d^
	PB	33^d^	1.80^a^	5.93^a^	2.15^b^	91.9^f^

*Values on the same column with different letters indicate significant differences (p<0.05). The letters “ab” indicate that the mean is not significantly different from those with the letter “a” and those with the letter “b”. CP = Cull potato waste; PP = peel potato waste; PB = pine bark waste.

Total C followed the same trend as that of fixed C for all biochars, where the concentration increased with increasing pyrolysis temperature across all feedstocks (Table 4). The PB biochar had greater total C concentration than those from potato wastes at both pyrolysis temperatures (Table 4). Total N content was decreased significantly (p<0.05) by increasing pyrolysis temperature from 350 to 650°C in the potato wastes, but not in PB. The PB biochar had higher (p<0.05) C/N than that of potato wastes at each pyrolysis temperature (Table 4). Biochars produced at higher pyrolysis temperature had significantly lower (p<0.05) O, O/C, H, and H/C. At 350°C, PB had higher O and O/C than CP and PP, while at 650°C CP had higher O concentration and O/C than PP and PB. There were no differences in H and H/C between biochars at each temperature.

**Table 4:** Ultimate results of the biochars.

Pyrolysis	Feedstock	Total	Total	C/N	O	O/C	H	H/C
Temperature		Carbon	Nitrogen					
(°C)		(%)	(%)					
350	CP	66.1^c^	2.19^d^	30.2^ab^	17^d^	0.257^e^	4.05^b^	0.0613^bc^
	PP	60.8^a^	2.49^e^	24.5^a^	13.7^c^	0.225^d^	3.83^b^	0.0629^c^
	PB	70.3^d^	0.346^a^	203^d^	24.7^e^	0.351^f^	3.78^b^	0.0538^b^
650	CP	71.5^d^	1.45^b^	49.3^c^	9.74^b^	0.136^c^	1.74^a^	0.0244^a^
	PP	63.8^b^	1.84^c^	34.7^b^	6.24^a^	0.0977^b^	1.73^a^	0.0272^a^
	PB	90.1^e^	0.367^a^	245^e^	5.09^a^	0.0565^a^	2.19^a^	0.0243^a^

*Values on the same column with different letters indicates significant differences (p<0.05). The letters “ab” indicate that the mean is not significantly different from those with the letter “a” and those with the letter “b” (similarly for “bc”). CP = cull potato waste; PP = peel potato waste; PB = pine bark waste

### Selected chemical properties of the biochar types

Biochar pH significantly increased with increasing pyrolysis temperature, with biochar from potato waste having higher than that from PB (Table 5). At 650°C pyrolysis temperature, the pH values were in the order PP > CP > PB. Potato waste biochar types all had alkaline pH values, while the PB biochar produced at 350°C had acid pH. The pH values in KCl (and in water) ranged 4.8–10.3 (6.7–11.1) for biochars produced at 350°C and 9.1–12.4 (9.1–12.6) for those at 650°C.

**Table 5:** Selected chemical properties of the biochar types.

		pH		P	Exchangeable cations				
Pyrolysis temperature	feedstock	(KCl)	(H_2_0)		K	Ca	Mg	CEC	CCE %
(°C)				mg kg^-1^	cmol_c_ kg^-1^			
350	CP	10.3^c^	11.1^d^	712^c^	15.9^c^	1.47^bc^	7.58^c^	57.7^d^	11.5^b^
	PP	10.1^c^	10.7^c^	194^b^	11.9^b^	2.39^d^	7.35^c^	31.0^c^	9.43^ab^
	PB	4.78^a^	6.65^a^	0.0^a^	0.92^a^	0.64^a^	0.340^a^	3.25^a^	8.01^a^
650	CP	11.6^d^	12.1^e^	1077^d^	13.7^bc^	1.07^ab^	2.95^b^	56.0^d^	17.5^c^
	PP	12.4^e^	12.6^f^	1147^e^	10.1^b^	3.48^e^	3.24^b^	10.2^b^	19.68^c^
	PB	9.10^b^	9.14^b^	0.0^a^	2.55^a^	1.71^c^	0.188^a^	2.82^a^	9.43^ab^

*Values on the same column with similar letter indicates a non-significant difference (p<0.05) and with different letters indicates a significant difference (p<0.05). The letters “ab” indicate that the mean is not significantly different from those with the letter “a” and those with the letter “b” (similarly for “bc”). CEC = cation exchange capacity; CCE = calcium carbonate equivalent. CP = cull potato waste; PP = peel potato waste; PB = pine bark waste

Extractable P increased with increasing pyrolysis temperature, with potato waste biochars having higher (p<0.05) concentrations than PB, at both pyrolysis temperatures. The extractable P in CP biochar was significantly (p<0.05) higher at 350°C (712 mg kg^-1^) and lower at 650°C (1077 mg kg^-1^) than those from PP (194 mg kg^-1^ at 350 and 1147 mg kg^-1^ at 650°C). Potato waste biochars had higher (p<0.05) ammonium acetate extractable K than PB, with no significant differences (p >0.05) between the pyrolysis (350 and 650°C) (Table 5)

Ammonium-acetate extractable Ca in PP biochar was 2.4 cmol_c_ kg^-1^ at 350°C and 3.5 cmolc kg^-1^ at 650°C, with no significant changes with pyrolysis for CP.

Increasing pyrolysis temperature from 350 to 650°C led to a decrease in Mg for PP and CP, with no change for PB. The trend of CEC was CP>PP>PB at both pyrolysis temperatures. The CEC was significantly decreased by increase in pyrolysis temperature (350 to 650°C) for PP, with no effect on CP and PB. Potato waste biochars had higher CCE (p<0.05) than PB, with no differences in CCE between potato waste biochars at each pyrolysis temperature.

### Biochar surface functional groups and physical structures

**Fig 1:**
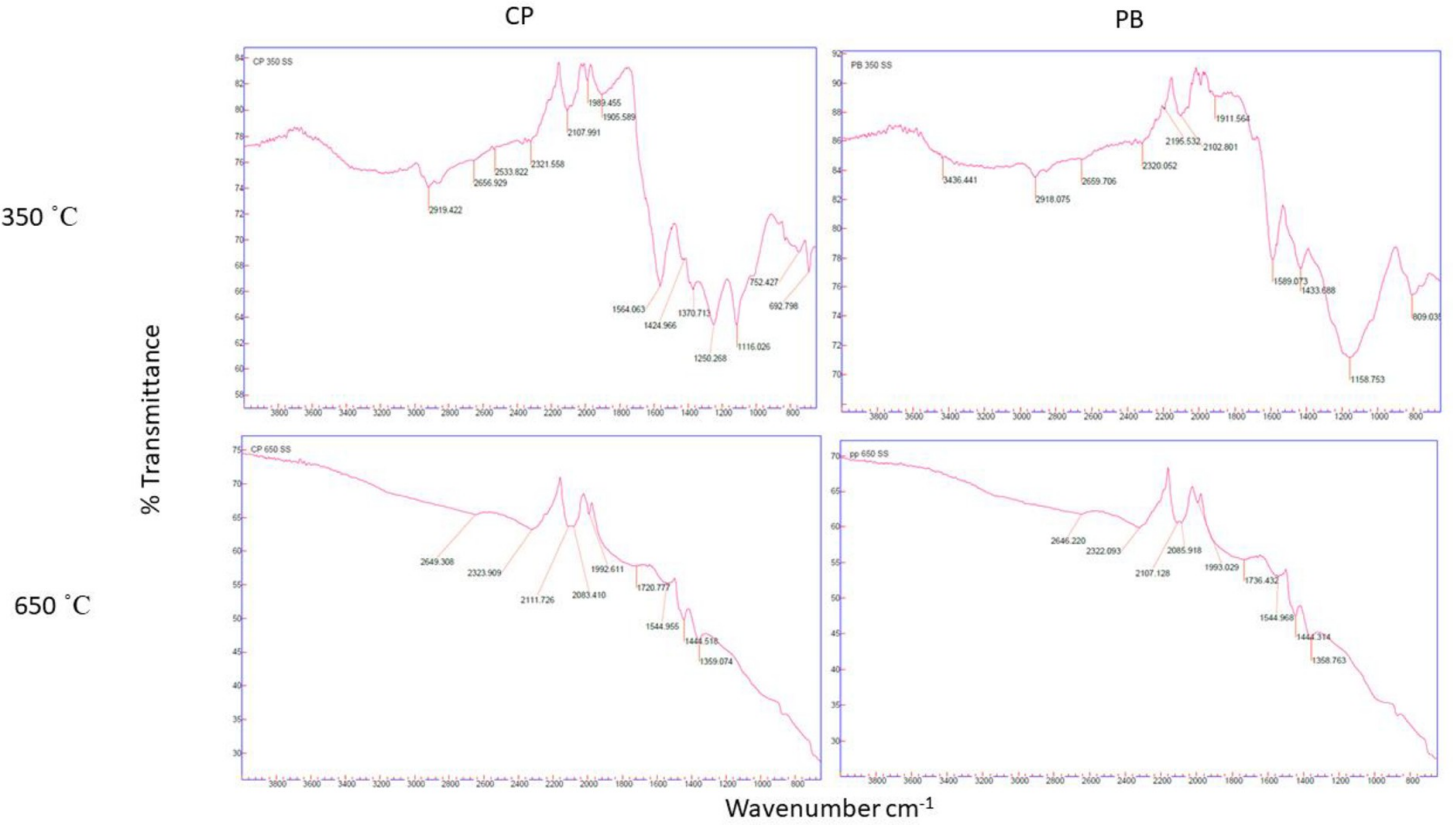
FTIR spectra visible of 4 studied biochars at different pyrolysis temperature using FTIR. CP = cull potato waste; PB = pine bark.

The chemical functional groups of the biochar types are shown in Table 6 and Fig 1. The C = C-C stretching, C = C bending of aromatic C and C-H bending of aromatic C functional groups were present in all biochars irrespective of feedstock and pyrolysis temperature. The heterocyclic amine functional group were only present in potato waste biochars pyrolysed at 350°C. The O-H stretching of carboxylic acid and metal carbonyl functional group was persistent for both pyrolysis temperatures for potato waste biochars, but not for PB pyrolysed at 650°C and 350°C, respectively. The aliphatic C-H stretch functional group only occurred in CP and PB pyrolysed at 350°C. The alkyne functional group was persistent throughout the pyrolysis temperature range for CP and PB, but not PP where it only occurred at 650°C. The aromatic C functional group only occurred in PB produced at 650°C. The stretching aldehyde functional group occurred in biochar from potato waste and PB at 650°C and 350°C pyrolysis temperature, respectively. The carbonate ions functional group occurred at both pyrolysis temperatures for CP, at 650°C for PP and at 350°C for PB. The O-H bending of phenols functional group were present at both pyrolysis temperatures for potato waste biochars. The C-O stretch functional group only occurred in PB, while the ether bonds–C-O-C and sulfate ions functional groups occurred in potato waste biochar pyrolysed 350°C. The phosphate ions functional group occurred in PP pyrolysed at 350°C and PB pyrolysed at 650°C.

**Table 6:** FTIR Spectra visible for biochars from potato waste and pine bark [36].

Frequency,	Functional group	CP		PP		PB	
Wavenumber (cm^-1)^		350 ˚C	650 ˚C	350˚C	650 ˚C	350˚C	650 ˚C
3490–3430	Heterocyclic amine	3436.44		3344.11			
3000–2500	O-H stretching of carboxylic acid	2595.38	2649.308	2886.35	2646.22	2659.71	
2950–2850	aliphatic C-H stretch	2919.42				2918.08	
2260–2100	Alkyne	2107.99	2111.726		2107.128	2106.62	2149.17
2100–1800	Metal carbonyl	1947.52	2038.011	2051.48	2085.918		2086.94
2000–1900	C = C-C stretching	1903	1992.611	1906	1993.029	1911.56	1989.9
2000–1750	Aromatic ^a^						1797.53
1740–1690	Stretching aldehyde		1720.777		1736.432	1710	
1700–1500	C = C bending of aromatic C	1564.06	1544.955	1563.33	1544.968	1589.07	1563.74
1490–1410	Carbonate ions	1424.97	1444.518		1444.314	1433.69	
1410–1310	O-H bending of phenol	1370.71	1359.074	1392.02	1358.763		
1300–1000	ether bonds -C-O-C	1250.27		1247.56			
1150–1050	C-O stretch					1148.78	
1130–1080	Sulfate ions	1116.03		1111.65			
1100–1000	Phosphate ions			1012.42			1009.71
860–680	C-H bending of aromatic C	722.613	840	756.926	840	809.035	799.303

*CP = cull potato waste; PP = peel potato waste; PB = pine bark waste. The letter a indicates assignment to combination bands

**Fig 2:**
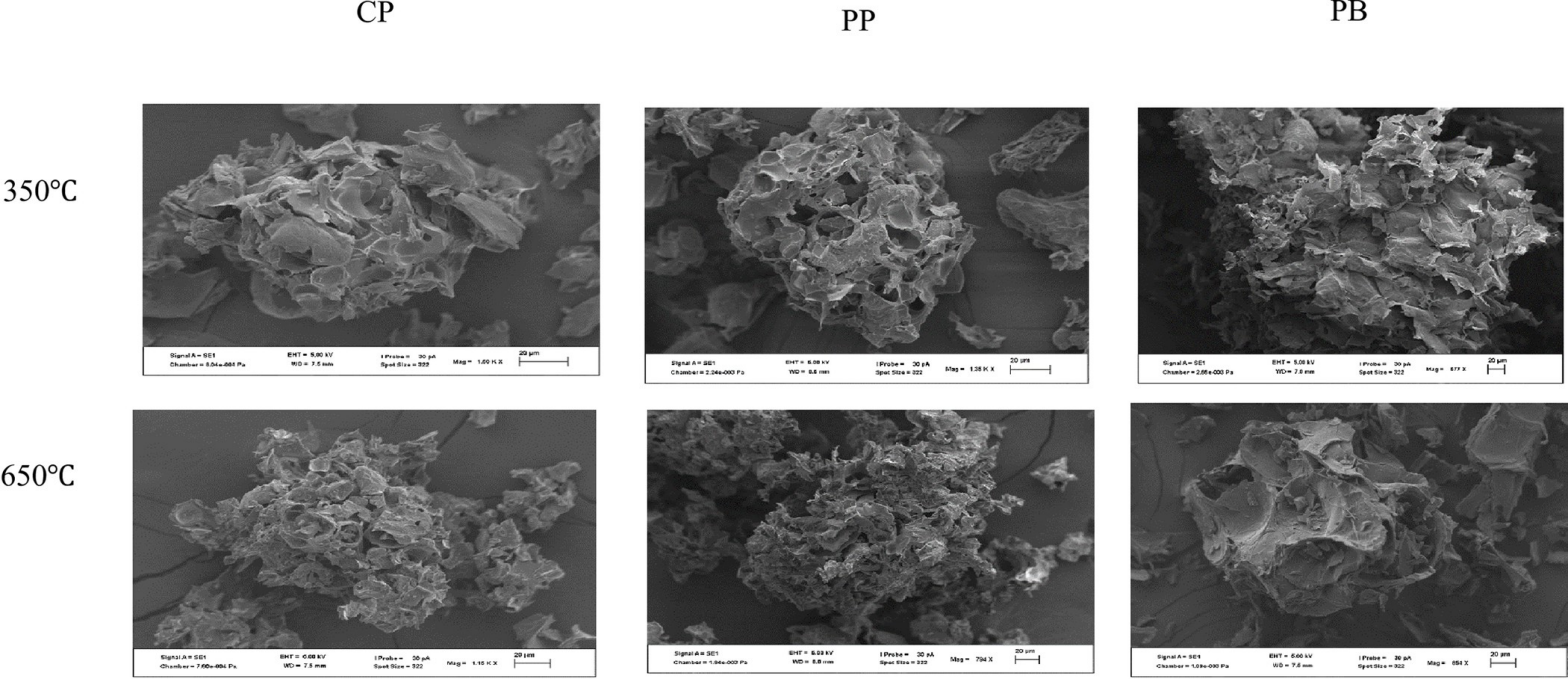
Morphological comparison of 6 studied biochars at different pyrolysis temperature using Scanning Electron Microscopy. CP = cull potato waste; PP = Peel potato waste; PB = pine bark.

The electron-microscope images indicated that biochar external morphology was highly affected by pyrolysis (Fig 2). Increasing pyrolysis temperature led to a considerable increase in pores, particularly for CP and PP biochars.

### Acid neutralisation

**Fig 3:**
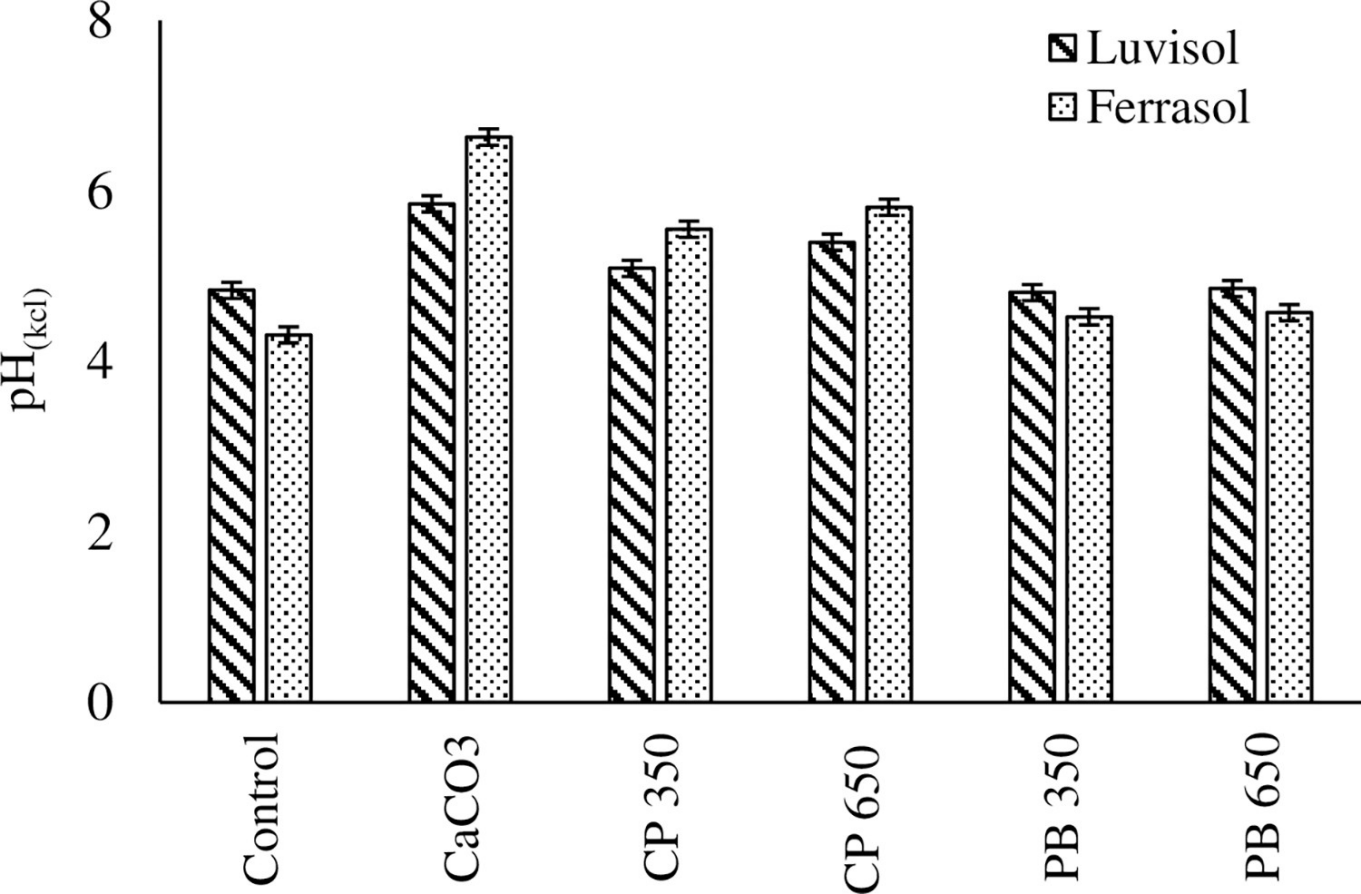
pH(KCl) values during a 10 day incubation of soils from luvisol and ferralsol amended with CaCO_3_ = calcium carbonates and biochars from cull potato (CP) and pine bark (PB) produced at varying pyrolysis temperatures. Lime applied at recommended rate. CP 350 = Cull potato biochar at 350°C; CP 650 = Cull potato biochar at 650°C; PB 350 = Pine bark biochar at 350°C; PB 650 = Pine bark biochar at 650°C. The vertical error indicates LSD (P<0.05).

Application of biochar significantly increased soil pH compared to the control, except for PB biochars, which showed no effect in the Luvisol (Fig 3). For the Ferralsol, addition of PB biochar had similar effects on soil pH, which were higher than the control and lower than the positive control (CaCO_3_). In the Ferralsol, CP biochar at 350°C had similar effect to CP 650°C applied in Luvisol. Addition of CP 350°C in Ferralsol had similar effect with the positive control applied in the Luvisol. For each soil type, all the biochar treatments had lower pH than the positive control.

## Discussion

Characterization of biochar provides a clear indication of significant differences in the composition of materials produced from different feedstocks at the same temperatures [37]. Differences in the biochar yield also reflected differences in the feedstock properties. For example, PB biochar produced at both 350°C and 650°C showed higher yield than the potato waste biochars due to higher thermal stability as a result of the rigid and compact structure of lignin [38]. This is similar to Gani and Naruse [39],Nanda et al. [40] who reported high biochar yield from biomass components with high lignin content. Pyrolysis of potato waste resulted in high mass loss due to the high cellulose content in the biomass [41, 42]. The lower yield in PP and CP than PB biochar could be explained by release of water vapour and thermal decomposition of lignocellulosic components and losses of C as carbon monoxide. This is consistent with several reports on biomass pyrolysis [22, 43, 44] and correspond with the results of the FTIR which shows losses of functional groups and the restructuring of the C groups (Table 6). The higher yield of the PP biochar relative to CP could be due to presence of inorganic compounds as suggested by the high ash content (Table 3). The decrease in moisture content with pyrolysis temperature is due to dehydration and consequent removal of O-H containing functional groups (Table 6).

The reduction in volatile matter at higher pyrolysis temperature (Table 3) was attributed to losses in low molecular functional groups such as aliphatic compounds as pyrolysis temperature increases, prompting aromatisation. Nguyen et al. [45] reported similar trend for biochars obtained from corn stover. The potato waste biochars 650°C had higher VM than PB owing to differences in cellulose, hemicellulose and lignin [46]. Sun et al. [42] reported that materials with high cellulose content produce biochar with high VM. Volatile matter affects material stability [17], N availability and inevitably plant growth [47]. According to the study by Zimmerman [48], biochars with VM of 40% showed a decomposition of 100 years of less than 10%. The higher VM in potato waste biochar maybe beneficial as a source for labile C for microbial communities [47] but could have negative implications for C storage due to positive priming effect.

The increase in ash content with increasing pyrolysis temperature (Table 3) could be due to accumulation of inorganic compounds (calcium carbonates, potassium silicate, iron and other metals) in the biochar [45]. Ash is the remaining solid after oxidation of all organic elements (C, H, and N) [14]. Low ash content of PB biochar compared to that of potatoes is consistent with Domingues et al. [14]; Sun et al. [42]; Nguyen et al. [45] who compared wood biochar and agricultural biomass. High ash content may be influenced by the nutrient concentration in the biomass [49].

The increase in fixed C with increasing pyrolysis temperature was attributable to losses in VM and the results were consistent with previous studies on biochar types [44, 50]. The higher fixed C in PB biochars compared to potato waste biochars, was ascribable to higher lignin content. Ash content acts as a heat resistant component [17] consequently hindering organic compound degradation and formation of aromatic structures. This can explain lower fixed C for potato biochar compared to PB. Similarly, Mimmo et al. [43]; Nguyen et al. [45] reported a negative correlation between ash content and fixed carbon. Biochars with a FC greater than 35%, limits their ash to be below 30% [17], this is however in line with our findings.

The increase in C composition with increasing pyrolysis temperature (Table 4) may be due to the intensified magnitude of polymerization producing a condensed aromatic carbon structure [14, 51]. Similar results were reported for biochars produced from miscanthus [43] and woody biochars [21]. Our findings could also be supported by the loss of oxygenated groups and H (Table 4), suggesting breaking down of weak bonds in biochars [52]. Similar to Enders et al. [17], PB biochar showed a larger increase in C composition relative to other biochars, due to the aromatic substructure, while the lower increase in C composition of potato waste biochar could be due to higher labile carbon. Biochars rich in C being produced at high temperatures can benefit carbon sequestration [45] due to resistance to microbial decomposition [13, 53]. Soils with very low organic material could benefit through the addition of biochar considering their high C content. This approach could benefit famers since it could be a convenient way of increasing soil organic carbon. Pine bark biochar produced at 650°C could be used to sequester carbon considering its high C content applying a recommended rate of 22.5-ton ha^-1^ of biochar.

Nitrogen composition of potato biochars was greater at 350°C pyrolysis temperature compared to pine bark, due to the enrichment of N containing heterocyclic compounds (Table 6) from higher N in the feedstock. However, the higher pyrolysis temperature of 650°C decreased N composition in CP and PP biochars, which could be explained by the volatilization of NH_3_ and N containing volatile compounds [54]. Literature indicates that N content usually decreases with temperature ranges of 500–800°C [55]. When applied to the soil the potato waste could supply more than 120kg N ha^-1^ when applied at 1% (kg N ha^-1^) equivalent to 285 (CP), 381 (PP), 49 (PB) feedstocks, 335 (CP), 412 (PP), 49 (PB) biochars at 350°C and 205 (CP), 291 (PP), and 41 (PB) biochars at 650°C. Consistent with Koetlisi and Muchaonyerwa [18] PB biochar showed no changes in N content.

The pine bark biochar had higher C/N ratio than potato waste biochars, which was in agreement with previous reports [42, 45]. The high C/N ratio could lead to increased N immobilization by microbes in the soil. This may occur due to recalcitrant C or the present of heterocyclic C [56]. The H/C and O/C decreased with the increase in pyrolysis temperature, owing to losses of O, H and polar surface functional groups hence increasing C content [57]. The general decrease in both O and H suggests similar condensations reactions for pine bark and potatoes waste. In this study, all the biochars were in the range H/C < 0.6 and O/C < 0.4 appropriate for sequestering carbon [58]. Biochars with O/C range (0.2–0.6) similar to those produced at 350°C in the current study are believed to have a half-life of 100–1000 years, and for O/C < 0.2 as the ones produced at 650°C in the current study are suggested a half-life greater than 1000 years [58].

Enders et al [17] expanded on Spokas [58] theory by including volatile matter, as also having influence in carbon sequestration. Volatile matter above 80% shows no carbon storage potential, VM less than 80% and O:C ratio greater than 0.2 or H:C ratio greater than 0.4 indicates moderate carbon storage potential; and VM less than 80% and an O:C ratio less than 0.2 or H:C less than 0.4 indicates high sequestration potential. The low O/C signifies structural arrangement of the aromatic rings making the biochar more stable [50]. The high O/C for CP at 650°C indicates presence of more functional groups in the biochar [44]. The conversion of waste to biochar would be a viable method for carbon sequestration and increasing soil organic carbon that will persist in soils for many years.

The rise in pH with increasing pyrolysis temperature (Table 5) was in line with Enders et al. [17], Walter and Rao [38] who reported alkaline biochars with increasing pyrolysis temperature. This is associated with an increase in salts in ash content, calcium carbonates equivalent (CCE) and loss of acid surface functional groups leaving oxygen functional groups [20]. Biochar from potato waste showed significantly higher liming potential than biochars from PB. This is shown by higher CCE and high pH [12] values for potato waste biochar relative to pine bark. Such high pH values have been previously seen in literature ranging from slightly acidic (4) to highly alkaline [13], depending on the feedstock and pyrolysis temperature [59, 60] and are supported by the presence of carbonates in the FTIR (Table 6) and consequent high CCE. Similar findings were reported for tomato biochar [61]. Application of potato waste biochars in an acidic soil increased soil pH while PB biochars application at similar rates of CCE did not influence the soil pH. The differences in the liming potential of the treatments could be the dissimilarities in the kinetic dissolution of alkaline salts in the ash of the biochars [30]. Also, the dissolution of some alkaline salts in soils make take longer than 10 days and differ. Another possibility for the increase in soil pH is the presence of the negatively charged functional groups in the potato waste biochars (Table 6) which bind H^+^ in the soil solution. The increase in soil pH following potato waste biochar addition could be in line with CCE and the inherently high pH, since Ca^2+^ displace the H^+^ and Al^3+^ and the H^+^ is neutralised in solution [62]. Similar results were reported for biochars produced from rice hull [63] and attributed to high alkalinity.

It is therefore worth noting that remediating acidic soils should not be evaluated solely by pH values, hence liming value which is affected by ash content should also be considered. Smallholders are facing a challenge of remediating acidic soils, due to high lime costs. Thus, using CP 650°C biochar as an alternative could be advantageous to small farmers as it will be more economical than limestone, however, additional lime could still be required due to limited quantities of biochar. Ameliorating an acidic soil raises pH thus improving nutrient availability, especially P, and microbial activity in the soil. Its application based on 10t c/ha results in under-liming in acidic soils.

High extractable P observed for potato waste biochar can be explained by high ash content and consequent increase on soil pH. Moreover, phosphorus is not lost via volatilisation, particularly at pyrolysis temperatures below 700°C [37]. However, high extractable P does not coincide with the FTIR results (Table 6), which shows a lack of phosphate. The available P may be partially bonded to -O- (phytic acid) in the carboxylic group or other functional groups (COO). The other reason could be P precipitating with Ca^2+^ forming an apatite, commonly observed at high pyrolysis temperature [64] owing to biochar being alkaline and having high carbonates ions (Table 6). The reason for the phosphate functional only at 350 but not at 650°C for PP biochar, and none in CP biochars, is not clear. From the agricultural point of view, application of potato waste biochars can increase the composition of available P (mostly in acidic soils) due to their liming ability. Application rates (kg P/ha) at 1% application rate were calculated for feedstocks CP, PP and PB (2.12, 1.13, 0.28) and their biochars pyrolysed at 350°C (10.9, 3.2, and 0) and 650°C (15.2, 18.2, and 0). This was done assuming that P will not be fixed. Based on this assumption, none of the materials would be sufficient to reach 60–100 kg P/ha. The use of biochar for available P with additional chemical P fertiliser could be an alternative as compared to feedstocks, since they are slower releaser of nutrients [65]. Feedstocks contained lower available P and thus they will require higher application rates compared to their biochars, so conversion of feedstock to biochar is a good alternative.

The higher levels of K and Mg in the potato-based biochars (Table 5) compared to pine bark are consistent with Nguyen et al. [45] who reported high K and Mg for plant-based biochars in comparison to wood biochars. The decrease in Mg with increasing pyrolysis temperature for PP and CP is due to the higher ash at 650°C which may mean that the Mg is in the form of carbonates, which are not extractable with ammonium acetate used when comparing to at 350°C. This is supported by the higher pH at higher pyrolysis temperature, due to release of hydroxide ions upon reaction of the carbonates with water. Considerably high K content of CP and PP biochars than that of pine bark may be due to high concentration of such elements in the feedstocks [7, 10]. However, CP and PP feedstock had higher K content relative to its biochar suggesting a slow release of K during pyrolysis. Johansen et al. [66] also recorded a decrease in K with pyrolysis temperature. This can be explained by K being bounded to the carbonyl functional groups [67] and also forming a stable compound (K_2_CO_3_) [68] and cannot be extracted using ammonium acetate method. Nonetheless, the high available K content in the feedstock doesn’t make its a suitable K supplement, since organic wastes are susceptible to nutrient leaching when applied directly to the soil [69]. Henceforth, CP and PP biochars could be a great substrate to add on soil as a source of K and could replace conventional sources of K. Biochar from non-woody material shows higher CEC values compared to wood [45]. This trend was observed in the current study, with pine bark having lower CEC compared to potato waste biochar. Increase in pyrolysis temperature decreased CEC, possibly due to degradation in volatile organic compounds and acidic functional groups (-COO- and -O-) associated with the negative surface charge biochar [70].

Differences in infrared spectra reflected water loss, organic matter combustion, and concentration of mineral components that resulted from the heat [71]. The bands assigned to C-H stretching markedly decreased due to degradation and dehydration of cellulosic and ligneous components. The loss of ether bonds at high pyrolysis temperature indicated loss of polysaccharides during pyrolysis which led to increased aromatic structures [72]. Low pyrolysis led to the increase in the intensity of carboxylic group while carbonyl group increased with increase in pyrolysis temperature, owing to decomposition of carbohydrates [22]. This enhanced condensation of biochar organic compounds. The findings were in line with that of Jindo et al. [21]. For PB, most functional groups were lost due to pyrolysis temperature while potato waste retained more functional groups because of ash content. Potato waste biochars produced at 650°C still contained weak functional groups, suggesting that it will increase microbial activity attributable to labile C added by the biochars when applied to the soil. The increase in pores at low pyrolysis temperature might be associated with the decomposition of carbohydrates.

## Conclusion

Potato waste biochars have lower yield and fixed carbon and higher ash content, nutrient content and volatile matter compared to those from pine bark. Potato waste biochars also have higher higher K composition, available P, pH, and CCE, especially at higher pyrolysis temperature. The incorporation of potato waste biochar (CP) at 650°C increases soil pH, which reduces soluble iron and aluminium and positive charges on oxides of iron and aluminium, thereby reducing P fixation and increasing its availability in acidic soils. Pine bark biochars had high C/N, FC, C, and low O/C, H/C and nitrogen with increasing pyrolysis temperature. Pine bark biochars mainly produced at 650°C can sequester carbon in the soil due to increased stability and aromaticity. The findings of this study confirm the view that, feedstock acts as a primary factor constraining biochar characteristics, while pyrolysis temperature acts as a modifier, influencing the physico-chemical properties and increasing the biochars’ aromatic character. Potato waste biochars have high agronomic value and should be tested for their ability to supply K and increase P availability in soils based on their liming potential. Moreover, understanding the effects of adding these biochars in near neutral and acidic soils on pH, CO_2_ emission, mineral N, available P, and available K is also required.

## Supporting information

S1 Data (XLSX)
